# Resolutions of Respect to Dr. Alonzo Boice

**Published:** 1898-08

**Authors:** 


### Obituary.

### RESOLUTIONS OF RESPECT TO DR. ALONZO BOICE.

At a stated meeting of the Odontological Society of Pennsylvania, held March 12, 1898, the following resolutions concerning the death of Dr. Alonzo Boice were read and adopted:

"Whereas, In the death of Dr. Alonzo Boice this Society has lost a public-spirited, generous, and devoted member;

"*Resolved*, That the members of the Odontological Society of Pennsylvania, of which the deceased was an active member, do hereby express the sense of their loss suffered in the death of Dr. Boice, and of their appreciation of his services to this Society and to the dental profession; and be it further

"*Resolved*, That these resolutions be incorporated and published in the proceedings of this Society, and a copy of the same be forwarded to his family.

"I. N. Broomell,
"Geo. W. Warren."

